# Validity of the dental operating microscope and selective dentin removal with ultrasonic tips for locating the second mesiobuccal canal (MB2) in maxillary first molars: An in vivo study

**DOI:** 10.4317/jced.59347

**Published:** 2022-06-01

**Authors:** Liliana A. Camacho-Aparicio, S. Aída Borges-Yáñez, Daniel Estrada, Minerva Azcárraga, Reneé Jiménez, Ricardo González-Plata-R

**Affiliations:** 1 Dental Public Health Department, División de Estudios de Posgrado e Investigación, Facultad de Odontología, Universidad Nacional Autónoma de México, Ciudad Universitaria, Av. Universidad 3000, Del. Coyoacán, C.P. 04510, México City, México

## Abstract

**Background:**

Several investigations have determined whether the use of a dental operating microscope (DOM) in combination with selective dentine removal with ultrasonic tips increases the percentage of location of the Mesiobuccal 2 (MB2) root canal in maxillary first molars (MFM). However, these studies did not report the performance of in vivo measurements with the comparison with a gold standard. The aim of this study was to determine the validity of the DOM and selective dentin removal with ultrasonic tips to locate the MB2 root canal in MFM using Cone Beam Computed Tomography (CBCT) as the gold standard.

**Material and Methods:**

The initial sample size was 91 patients, but 7 were excluded, so the sample size was 84 patients who signed the informed consent. Inclusion criteria: MFM indicated for root canal treatment. An expert blinded observer identified the MB2 in the CBCT. Two standardized examiners (Kappa=91%) performed the clinical assessment in three stages: Stage 1, canal location with an endodontic explorer and a mirror; Stage 2, use of DOM and Stage 3, use of DOM plus selective dentine removal with ultrasonic tips. The validity of each stage was calculated.

**Results:**

The prevalence of MB2 using CBCT was 79%, by clinical location was 68%. Sensitivity was 79%, 82%, 86% for stage 1, 2 and 3, respectively. Specificity and Positive Predictive Values were 100% for all methods. Negative Predictive Value was 56%, 60%, 67%, respectively. Positive Likelihood Ratio tends to infinity for all methods, Negative Likelihood Ratio= 0.21, 0.18 and 0.14 and Accuracy= 83%, 86% and 89%, respectively.

**Conclusions:**

The use of DOM with selective dentine removal with ultrasonic tips is the most valid method for locating MB2 canal in MFM. There was an increase in the location of the MB2 root canal with the DOM and ultrasonic tips, which definitely help the clinician.

** Key words:**Cone beam computed tomography, microscopy, second mesiobuccal canal, sensitivity and specificity, validity.

## Introduction

It is known that the mesiobuccal root of maxillary molars is one of the most ramified branches of all human teeth (1). The presence of a second canal in the mesiobuccal root, mesiobuccal canal 2 (MB2) of the upper molars, has been studied in several investigations, one of de first studies evaluated 208 mesiobuccal roots of maxillary first molars, and MB2 was found in 51.5% (2). However, recent studies have found MB2 in much higher percentages, such as this study where they report that MB2 was located in 98% of 250 maxillary first molars analyzed by CBCT in Belgium (3).

The MB2 root canal has been found more often in *in vitro* studies than in *in vivo* studies. A review of the literature found that the mesiobuccal root presents two or more root canals in 57%, 60.5% in laboratory studies and 54.7% in clinical studies. This study reported that in *in vitro* studies, the MB2 root canal has been found to range from 25% to 93%, while *in vivo* studies, the localization rate ranges from 18.6% to 80.3% (4).

The lack of location (and treatment) of root canals can lead to a guarded prognosis. In treatment, teeth with nonlocalized root canals have a 4.38 times higher risk of having periapical lesions (5). In an investigation, the first maxillary molar was the tooth with the highest percentage of retreatment (6), as well as the tooth with the highest percentage of nonlocalized root canals (5).

Traditionally, root canal location methods have been based on mental images of the pulp floor anatomy and the tactile sensitivity of the operator since visibility is restricted. The use of dental operating microscope and illumination allows us to observe the differences in color between the dentin of the floor and the pulp chamber walls, facilitating the location of the canal. It has been indicated that the most important use of the microscope in nonsurgical endodontics is the location of complex root canals (7) and that MB2 root canal detection in upper first molars increases from 73.2% to 93% when using magnification (8). A recent clinical study found that the use of a microscope increases the percentage of MB2 canal localization from 26.67% to 77.78% in maxillary first molars (9). Another investigation reported that in 93% of cases, the MB2 root canal entrance was located without an operating microscope. The authors pointed out that the use of the surgical microscope is not critical for the localization of the MB2 canal, although the ability to negotiate it was facilitated, on the other hand, calibration of operators was not disclosed (10).

The MB2 root canal orifice is small and generally more difficult to identify than other canals. It can be found hidden under calcifications or dentin tissue and can be discovered by selective dentine removal with ultrasonic tips. This method has been shown to be more helpful in locating the MB2 root canal in a more conservative manner compared to the use of dental burs (11).

There is a strong emphasis in the use of cone beam computed tomography (CBCT) for the evaluation of the complex internal anatomy of teeth (12), and CBCT examination has been shown to be a valid method to detect the MB2 root canal in upper molars (13). One study reported that CBCT has a sensitivity of 96% and a specificity of 100% for the location of the MB2 root canal in endodontically treated upper molars (14). In a systematic review, the combined prevalence of MB2 was 70% using CBCT from the mesiobuccal root of 15,285 maxillary first molars (3). Today, the gold standard imaging technique to assess the presence of an MB2 canal in a clinical setting is CBCT (15).

Several investigations have determined whether the use of a dental operating microscope (DOM) in combination with selective dentine removal with ultrasonic tips increases the percentage of location of the MB2 root canal in the upper first molars (16-18). However, these studies did not report on the reliability of the measurements, questioning the validity of the results. A study involving 33 examiners found that the percentages of MB2 canal detection in maxillary first molars were 71.1%, 62.5% and 17.2%, using microscope, dental loupes, and no magnification respectively, in maxillary second molars were 36.1%, 40.5% and 20.0% in the same groups (19). Even when the examiners were calibrated, there were several variables, such as access through crowns, the experience of operators, the number of appointments or the clinician’s persistence in locating the root canal, which can increase the risk of bias. In addition, with a greater number of examiners, interexaminer reliability increases the variability of the measurements even when examiners are standardized (20). Conversely, some investigations did not present clear operational definitions for the location of the MB2 root canal (18,19,21) or the validity of the reported criteria measurements when a gold standard was used (16,21).

The reliability of the measurements, the calibration of the examiners, the performance of *in vivo* measurements, and the comparison with a gold standard increase the reliability and validity of studies aimed to identify the location of root canals in endodontics. However, there is no study aimed at validating the use of the dental operating microscope and selective dentine removal with ultrasonic tips for the location of the MB2 canal in upper first molars that meet these criteria.

The purpose of this study was to estimate the validity of the use of dental microscope and selective dentine removal with ultrasonic tips as methods for locating the MB2 root canal in the upper first molars using cone beam computed tomography (CBCT) as the gold standard.

## Material and Methods

This cross-sectional validation study was carried out according to the STARD statement to report studies of diagnostic accuracy (22). The research protocol was approved by the Research and Ethics Committee of the School of Dentistry, UNAM (CIE/0110//03/2017). This present study was carried out following the General Health Law on Research in Mexico and the principles of the Declaration of Helsinki.

The study population was first upper molars indicated for root canal treatment of patients between 18 and 50 years of age who attended the Endodontics Clinic at the Graduate Division, School of Dentistry, National Autonomous University of Mexico in the period 2017 to 2018. Teeth with resorption in the pulp chamber, immature apex, vertical fracture, or previous root canal treatment were excluded. Patients who met the selection criteria were invited to participate in the study, and the first 91 patients who agreed to participate and signed the informed consent form were included in the study. Participant recruitment took place from February 2017 to May 2018.

Sample size was calculated using a formula for sensitivity and specificity for diagnostic studies with the PASS 15 program. A power of 81% was used as the calculation assumption to detect a change in sensitivity from 0.8 to 0.9, and the significance level of the sensitivity test was 0.046. A sample size of 91 teeth was required, assuming a prevalence of the MB2 canal of 90%.

The variables were sex (male/female) and age (years), the tooth to be treated (right or left first upper molars), and the MB2 location methods: Stage 1: Direct vision, location of the canal with an endodontic explorer (DG-16 Hu friedy® Des Plaines, Illinois USA), a mirror and a hand file of size 10; Stage 2: Use of operating microscope (Carl Zeiss Opmi Pico); Stage 3: Use of operating microscope and dentin removal by ultrasonic tips (NSK® Nakanishi inc. Japan) The gold standard was CBCT analysis of each tooth to confirm the presence or absence of the MB2 root canal. Clinically, the MB2 root canal was considered located if it was possible to introduce a Dentsply Maillefer® K file # 8 or # 10 in the cervical and middle third of the root canal according to the estimated working length.

Each root canal treatment and clinical analysis was performed by two calibrated examiners, which were calibrated for the clinical location of the MB2 root canal, in three stages using 10 extracted first upper human molars placed on a typodont (Nissin® Kyoto Japan) and mounted on a simulator (Nissin® Kyoto Japan). The interexaminer reliability was calculated using Cohen’s kappa, obtaining values between 81% and 100%. One month later, the teeth were mixed, and all observations were repeated on each tooth by each examiner to calculate the intraexaminer reliability. Cohen kappa values between 88% and 100% were obtained.

**Figure 1:**
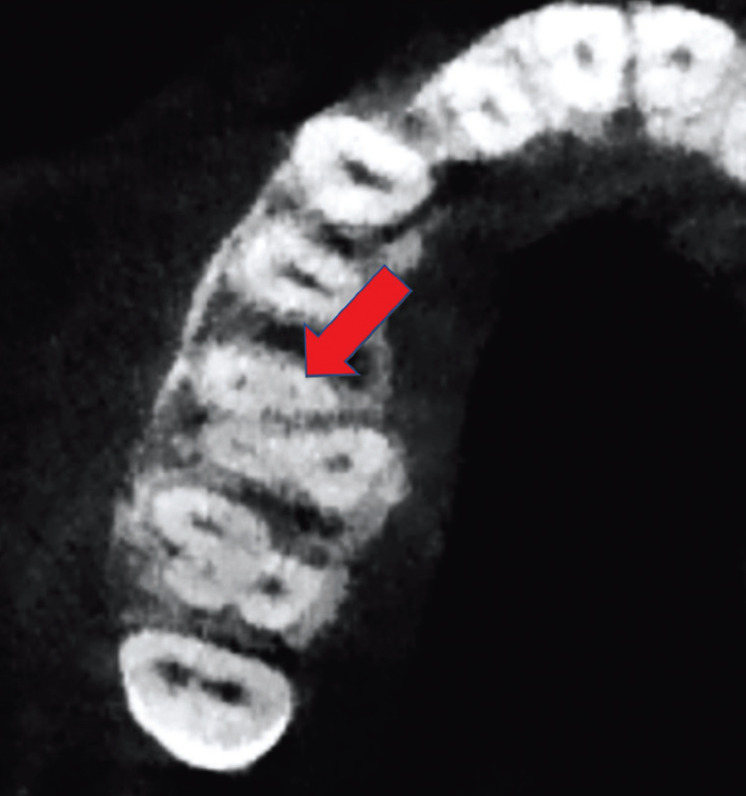
MB2 root canal location in a right maxillary first molar using CBCT.

Each patient underwent CBCT (NewTomVGi® Verona Italy tomograph, 0.3-mm voxel measurement). One expert performed the CBCT analysis of each tooth to confirm the presence or absence of the MB2 root canal and was blinded to the clinical presence of the root canal. The MB2 root canal was considered to be present when it was visible in the CBCT axial view of the coronal third of the root (Fig. 1)

This information was recorded and was provided to the clinical examiners after carrying out the clinical stages of the MB2 canal search. Each root canal treatment was performed by one of the examiners. The operator anesthetized the patient and performed the absolute isolation with dental dam and a #7 dental clamp (Hygenic® Akron, Ohio USA). The access into the pulp chamber was done with a #4 round carbide bur (SS White® New Jersey USA) and an endo Z bur (Dentsply® Tulsa, Oklahoma USA), the walls were smoothed with an E7D ultrasonic tip (NSK® Nakanishi inc. Japan), all the operative steps and fundamental access principles were followed according to the anatomy of each tooth. The pulp chamber was irrigated with 2.5% sodium hypochlorite (NaOCl). The mesiobuccal 1 (MB1), distobuccal (DB) and palatal (P) root canals were located using an endodontic explorer DG-16 Hu Friedy®. Subsequently, the operator proceeded to explore the pulp chamber in search of the MB2 root canal in three clinical stages, the same ones that were carried out in the standardization of the observers:

STAGE 1: Direct vision: Root canal location with a mirror, an endodontic explorer, and a file: The MB2 root canal search was carried out with the help of a DG-16 Hu-Friedy® endodontic explorer, a Hu-Friedy® #5 front view mirror and a Dentsply Maillefer® K file #8 or #10. The search for the root canal was carried out in a mesial position with respect to an imaginary line between the MB1 and P root canals, less than 3.5 mm palatally and 1-2 mm mesially from the MB1 root canal orifice.

STAGE 2: Use of a dental operating microscope: If the MB2 root canal was not located in stage 1, an additional exploration was performed with the use of a Carl Zeiss® Opmi Pico operating microscope. The approach was made with a 1.6 magnification factor, and the fine focus was performed using the Varioskop 100 variable focus adapter.

STAGE 3: Use of a dental operating microscope plus selective dentine removal with ultrasonic tips: If the MB2 root canal was not located in stages 1 and 2, selective dentine removal with an NSK® E7D ultrasonic tip was used to less than 3.5 mm between the MB1 root canal and the palatine canal, 1-2 mm mesial to the MB1 orifice and 2 mm depth, or where the MB2 root canal trace would be observed according to the particular anatomy of each tooth. The ultrasound Varios 350 NSK® was activated at power 4. Conservative removal was performed and explored with a DG-16 Hu-Friedy® explorer.

**Figure 2:**
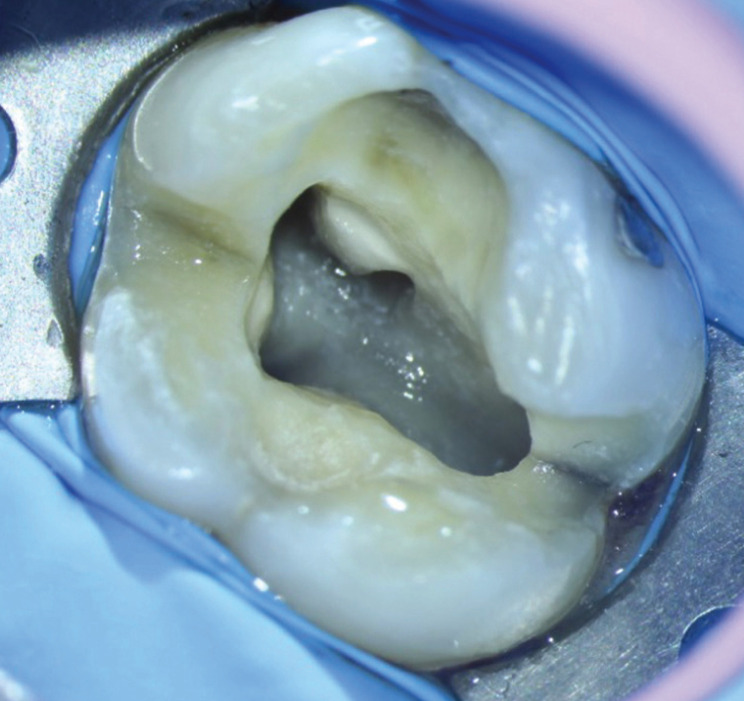
MB2 root canal location, photograph taken under microscope.

**Figure 3:**
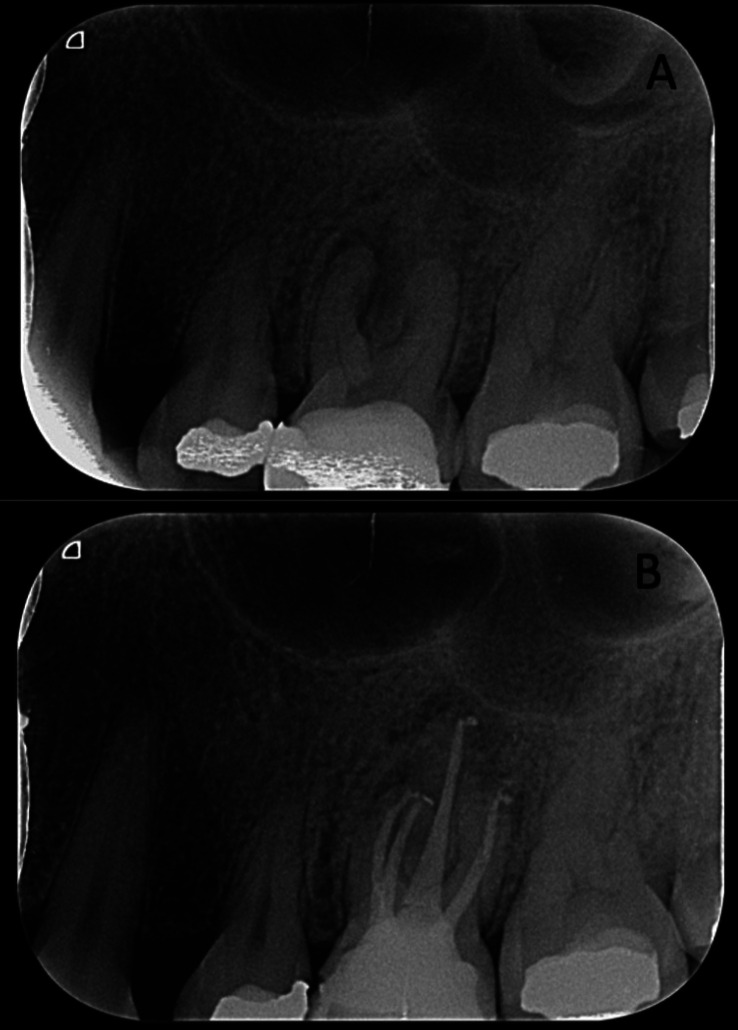
A) Initial and B) final radiographs of the root canal treatment in the maxillary left first molar with location of the MB2 root canal.

In any of the stages, the MB2 root canal was considered localized if it was possible to introduce a Dentsply Maillefer® # 8 or # 10 K file in the cervical and middle third of the canal according to the estimated working length measured with the scanner (Sidexis®Dentsply Sirona, Charlotte NC, USA). The location of the canal was confirmed using an electronic apex locator and radiographs to confirm the direction and presence of the canal, as well as photographs taken under a microscope using a camera (Canon Rebel T6, Tokyo Japan) (Fig. 2). The root canals were shaped and filled according to each clinical case (Fig. 3).

The general data of the patient, the tooth to be treated, and the presence or absence of the MB2 root canal in the clinic were recorded, if it was located, in which stage it was located and whether it was present or not by tomographic analysis. The data were analyzed using the statistical software Stata version 15.

Several measures were calculated to estimate the validity of each method, the probability of correctly identifying the MB2 root canal (sensitivity), the probability of correctly identifying teeth without an MB2 root canal (specificity), the probability that the MB2 root canal is present when it is detected (positive predictive value), and the probability that it is absent when it is not detected (negative predictive value), the ratio of the proportion of teeth with MB2 testing positive and the proportion of teeth without the canal also testing positive (positive likelihood ratio), and the ratio of the proportion of teeth that with MB2 testing negative and the proportion of teeth without the canal testing negative (negative likelihood ratio).

Finally, the total percentage of MB2 root canals correctly identified (accuracy) was calculated. The diagnostic accuracy measurements for each method were calculated against the gold standard and the 95% confidence intervals for each measurement.

## Results

The mean age of the patients was 35.7 ± 9.1 years, the maximum age was 50 years, and the minimum age was 19 years. Of the participants, 64.3% were women, and 91 patients were analyzed through CBCT; however, four were excluded for failing to follow with the study, and three were excluded because the MB2 root canal was located clinically but not in tomography; therefore, 84 maxillary first molars were included in the study. The power of this sample size was estimated, a total sample size of 84 (which includes 66 subjects with the MB2 root canal) achieves 66% power to detect a change in sensitivity from 0.8 to 0.9 and 98% power to detect a change in specificity from 0.8 to 0.999. The actual significance level achieved by the sensitivity test is 0.03 and achieved by the specificity test is 0.02. The prevalence of the MB2 root canal is 79%.

According to the location in the maxillary, 46 (54.8%) were located in the right upper quadrant, 66 MB2 root canals (78.6%) were detected by cone beam computed tomography (gold standard) analysis, and 57 (67.8%) were identified in the clinical stages of localization. Nine (13.6%) of the 66 MB2 root canals located in the tomographic analysis were not located clinically. Of the 57 clinically located MB2 root canals, 52 (91.2%) were detected in Stage 1 (root canal location using a front view mirror, a DG16 endodontic explorer and a K # 8 or # 10 Dentsply® file), only two cases (3.5%) were located in Stage 2 (MB2 root canal location plus the use of DOM), and three patients (5.3%) were located in Stage 3 (MB2 root canal localization and the use of DOM plus selective dentine removal with ultrasonic tips) (Table 1).

**Table 1:**
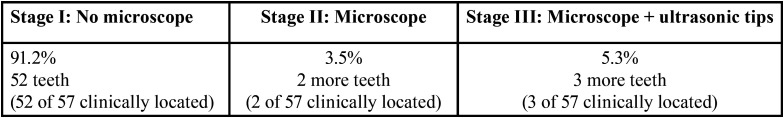
Percentages of MB2 clinical detection according to each clinical stage.

The results from Stage 1 indicate that the MB2 root canal had 79% probability of being detected using only a mirror, an endodontic explorer, and a file; however, the probability of not being present when the diagnosis was negative was 56%. The likelihood ratio of a positive test indicates that it was 78.8 times more likely to observe a positive result in a tooth with an MB2 canal than in a tooth with no MB2 canal. The likelihood ratio of a negative test indicates that it was 0.21 times less likely to observe a negative test in a tooth with an MB2 canal than in a tooth with no MB2 canal. Localization with this method had a great effect in increasing the probability of the presence of the canal, while the negative likelihood ratio was 0.21, indicating that the result of this method had a moderate effect in decreasing the probability of the presence of the canal. The overall accuracy was 83.3%, showing the general proportion of correct results of the test (Table 2).

**Table 2:**
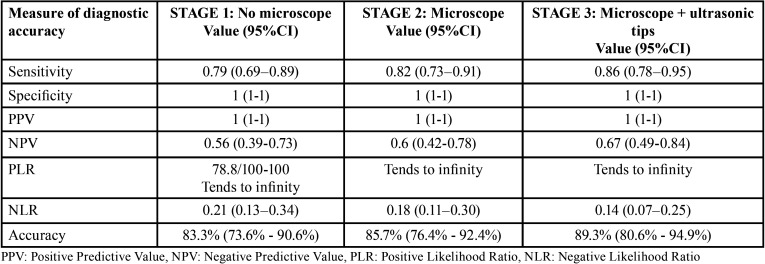
Measurements of the diagnostic accuracy of each MB2 root canal clinical location stage.

In Stage 2, the surgical microscope detected 82% of the MB2 root canals and 100% of teeth with no MB2 canal. The probability that the MB2 canal was not actually present was greater than in Stage 1. The positive likelihood ratio tended to infinity, indicating that the result of this method had a great effect in increasing the probability of the presence of the canal, while the negative likelihood ratio was 0.182, indicating that this method had a moderate effect in decreasing the probability of the presence of the canal. The overall accuracy was 85.7% (Table 2).

In Stage 3, the MB2 root canal had the highest probability of being detected (86%) when using a dental operating microscope plus selective dentine removal with ultrasonic tips. The positive likelihood ratio tended to infinity, indicating that this method had a great effect in increasing the likelihood of the presence of the canal, while the negative likelihood ratio was 0.136, indicating that the result of this method had a relatively high effect on the decrease in the probability of the canal presence. The overall accuracy was 89.3%, indicating the general proportion of correct results of the test (Table 2).

## Discussion

The accuracy of the three methods to identify the MB2 root canal in the upper first molars was high: 83.3% for the clinical location using a mirror, an endodontic explorer and a hand file; 85.7% with the help of a dental operating microscope; and 89.3% with the use of a dental operating microscope plus selective dentine removal with ultrasonic tips. The accuracy of the method using a microscope and ultrasonic tips increased by 6% compared to the location of the canal with mirror, endodontic explorer, and a file.

The prevalence of MB2 root canals identified by CBCT in this study (78.6%) was similar to Martins *et al*. (3) (73.8%) and was slightly higher than that reported in a systematic review (70%), including 15,285 maxillary first molars (15).

Regarding the stages of localization, Yoshioka *et al*. (16) and Alacam *et al*. (17) searched for the MB2 root canal by exploring the pulp floor in the same three stages or clinical phases as our study but using extracted teeth, which increased the probability of localization. In agreement with Alacam *et al*. (17), we found most MB2 canals in the first stage and 100% specificity in the three measurements. Without a microscope, with a microscope, and using a microscope plus ultrasound, they found sensitivities of 75%, 82% and 90%, respectively. These results are similar to those obtained in the present study (79%, 82% and 86%, respectively). Although a gold standard was used in Yoshioka’s study, the results of diagnostic precision measurements were not shown, and unlike the present study, they located most of the MB2 root canals in stage 3 using a microscope plus ultrasonic tips.

In another study, the MB2 root canals were found without a microscope in 93% of cases and 96% when using a microscope (10). In a similar study (21), MB2 root canals were found without a microscope in 51% while 63% were found when using a microscope (21). Both studies used a combination of first and second maxillary molars, only showed percentages of location with and without a microscope and did not calculate diagnostic accuracy measurements.

Similar to our study, Das *et al*. (23) and Sujith *et al*. (18) performed the localization of the MB2 canal in patients, evaluating the same clinical stages of localization as we did (Stage 1: use of mirror and explorer, Stage 2: use of a dental operating microscope and Stage 3: use of a microscope plus ultrasonic tips); however, neither study specified which gold standard was used and did not clearly report diagnostic accuracy measurements. Unlike our investigation, Sujith *et al*. (18) located only a few MB2 canals in Stage 1 and found only 12 canals in Stage 1 (20%). With the use of DOM, the MB2 canals were located in 21 more teeth (55%), and with the combined use of ultrasonic tips and DOM, the canals were located in 9 more teeth (70%) of a total of 60 first upper molars. MB2 root canals were detected by Das *et al*. (23) in 36%, 54% and 72% of the teeth in stages 1, 2 and 3, respectively. The probable reasons why clinicians in our study located most MB2 canals without a microscope or ultrasound could be due to having more clinical experience performing root canal treatment, and the fact that operators were calibrated allowing the location to be more detailed and comprehensive.

It is worth noting that in most of the studies, the results presented are percentages of location, without using the diagnostic accuracy measures (16,19,21,23). Diagnostic precision measurements are essential in these types of studies, as they help quantify the efficiency of the method at identifying subjects with or without a condition compared to a gold standard (24).

In the present study, a gold standard was used, and diagnostic accuracy measurements were calculated, the sensitivity increased by 7% and the accuracy by 6% between Stage 1 (a mirror, an endodontic explorer and a file) and Stage 3 (an operating microscope plus selective dentine removal with ultrasonic tips). We noticed that these devices also have the advantage of locating anatomical access cavities more precisely according to the anatomy of each tooth, with better illumination and more detailed visualization. The use of these localization methods is recommended; however, the increases in sensitivity and accuracy are not large enough for their use to be considered critical and essential for canal location.

Among the strengths of this study, we can point out the calibration of the two examiners, who achieved high values of inter- and intra-examiner reliability. In addition, specific criteria and clear operational definitions of each of the localization stages were used, reducing information bias and the variability of the measurements. When the examiners are not calibrated, some variables may not be controlled, such as access through crowns, the experience of the operators, the number of appointments or the persistence of the clinician in locating the root canal. All these increases the risk of information bias, as clinical opinions can vary among experts. Biased results can lead to improper recommendations about testing, negatively affecting patient outcomes (22).

In this study, the gold standard was clearly defined, and validity measures were calculated, which accurately indicate the probability that the location of the MB2 root canal is correct according to each of the localization methods. Another strength of this study is that a sample size was calculated, which helps us to have greater validity of the results. In the present study, a loss of 7 subjects was observed, which increases the probability of committing the type 2 error, however, the sensitivity of the study was not significantly altered for this reason. It is recommended that in subsequent studies the sample size be calculated assuming loss of subjects.

Among the limitations of the study, it can be noted that the voxel size (0.3 mm) of the cone beam computed tomography that was used was not the most appropriate since it was found that in three cases, MB2 was clinically located but was not visible on tomography. A similar situation was reported by Parker *et al*. (25); in three first upper molars, the MB2 root canal was located clinically but not on tomography, even when a Carestream 9000® tomograph with a voxel size of 0.076 mm was used. This indicates that although the use of CBCT is the most valid method to locate the MB2 canal in patients, the probability of not detecting it still exists; however, this may be due to calibration failures or variations in the use of the equipment. For future investigations, it would be advisable to repeat the calibration of the equipment and periodically reinforce operator standardization. On the other hand, in this study, CBCT was used as the gold standard, but we do not recommend using it in all cases. Similarly, Hiebert *et al*. (26) stated that exposing all patients to a tomographic scan before any treatment may not be appropriate, suggesting that when the MB2 root canal is not found clinically, taking CBCT can significantly increase its location.

The present study and the aforementioned studies found that the use of magnification (magnifying glass or microscope) and/or selective dentine removal with ultrasonic tips increases the percentage of location of the MB2 root canal in the upper first molar (8,16-18,21,23); therefore, the use of these devices is suggested to improve the prognosis in treatment.

## Conclusions

The use of a dental operating microscope in combination with selective dentine removal with ultrasonic tips is the most valid method to locate the MB2 root canal in maxillary first molars; therefore, its use is recommended. However, with the utilization of this method, the accuracy in locating the MB2 root canal increased by only 6% compared to localization with only a mirror, an endodontic explorer, and a hand file. Regardless of the method used to locate the MB2 root canal, in all investigations, there were always no localized canals due to the anatomical complexity of the root; therefore, more studies are needed to evaluate the findings of the present study with the prognosis of the root canal treatment.
